# Views of university students in Jordan towards Biobanking

**DOI:** 10.1186/s12910-021-00719-y

**Published:** 2021-11-13

**Authors:** Faisal Khatib, Dayana Jibrin, Joud Al-Majali, Mira Elhussieni, Sharifeh Almasaid, Mamoun Ahram

**Affiliations:** 1 Department of Physiology and Biochemistry, School of Medicine, The University of Jordan, Amman, Jordan; 2 School of Medicine, The University of Jordan, Amman, 11942 Jordan

**Keywords:** Biobanking, University students, Jordan, Research participation, Privacy, Informed consent, Medical school, Biomedical research, Genetic Research

## Abstract

**Background:**

Biobanks are considered primary means+ of supporting contemporary research, in order to deliver personalized and precise diagnostics with public acceptance and participation as a cornerstone for their success.

**Aims:**

This study aims to assess knowledge, perception, and attitudes towards biomedical research and biobanking among students at the University of Jordan.

**Methodology:**

An online questionnaire was designed, developed, and piloted. It was divided into 5 sections that included questions related to issues of biomedical research and biobanking as well as factors influencing the decision to participate.

**Results:**

Responses from 435 students revealed that 52.9% previously heard of biobanks. There was an overwhelming acceptance for participation in biomedical, genetic, and biobanking research. A blood sample was the most preferred for donation. Protection of privacy, informed consent prior to donation, approval of an ethics committee, and trust towards researchers were the most important factors associated with willingness to participate. On the other hand, the vagueness of the type of research performed on the biospecimens and the unavailability of general research results to the donor had a negative connotation. There was no clear agreement on the type of informed consent preferred by students, but to be contacted and informed of research results was preferred by the majority. Students also preferred the disposal of biospecimens and information when deciding to withdraw from participation.

**Conclusion:**

There is strong enthusiasm among students to participate in biomedical research and biobanking with all rights reserved thus providing hope for a very promising future in Jordan.

**Supplementary Information:**

The online version contains supplementary material available at 10.1186/s12910-021-00719-y.

## Background

Understanding the molecular etiology and distribution of specific diseases among groups of people of various ethnic backgrounds has necessitated the advent of biobanking. Biobanks are well established in high-income countries but are slowly emerging in low- and middle-income countries. The inclusion of low-income countries in biomedical research can advance multiple aspects of disease distribution, diagnosis, and treatment, particularly, considering genetic diversity [1, 2]. Jordan, located in the central region of the Middle East, is often a preferred place to receive excellent health care due to its advanced medical system and renowned physicians. The nation hosts a heterogeneous mixture of different and unique ethnic groups with relatively similar cultural backgrounds. Thus, data generated from biomedical research are expected to provide an insight regarding public health in the region [3].

Biobanking refers to the process by which biospecimens, along with associated data, are collected and stored in repositories under optimal conditions. These high-quality biospecimens may then be disseminated to researchers to identify disease biomarkers, for example, and to ultimately improve the understanding of health and disease [4]. Moreover, biobanks have played an important role throughout pandemics. A good case is the University of California at San Francisco’s AIDS Specimen Bank (ASB), which was founded in December 1982 in response to the early challenges of the AIDS epidemic. ASB provided investigators with high-quality biospecimens that contributed to the identification of the agents that cause AIDS and Kaposi's sarcoma [5]. With the COVID-19 pandemic defining the years 2020 and 2021, the whole world is witnessing the international efforts for vaccine and therapeutic development, which further highlights the importance of patient biospecimens [6].

For a biobank to be successful, a large and diverse number of individuals should be willing to participate. However, public participation is highly influenced by ethical, social, and legal challenges. Likewise, the issues of informed consent, privacy and confidentiality, return of results, and data-sharing are also controversies that need to be resolved [7]. Thus, the concept of biobanking needs to be publicly discussed and challenges in recruiting potential donors should be unraveled at the national level [8, 9]. Indeed, cultural-social norms of the community play an important role in whether the community is willing to donate and contribute to medical research.

Previously, approximately two-thirds of Jordanians expressed willingness to participate in biobanks with correlation to younger age [8]. The increasing number of university students in Jordan may influence other sectors of society. Hence, it is imperative to learn of their perception, knowledge, and attitudes toward biobanks. Not only that university students may be potential participants of biobanks, but they may also have an impact on the social acceptance of biobanks. The University of Jordan is the largest in Jordan encompassing 94 majors at the bachelor’s degree level, is located in a hub of the capital, Amman, and is attended by students of various demographic backgrounds. Herein, we targeted students at the University of Jordan in order to explore their knowledge, attitudes, and practice towards biomedical research and biobanking. In addition, other aspects of biomedical research and biobanking such as the main factors that influence student willingness to participate in and consent for biobanking research were assessed.

## Methods

### Study design and ethical approval

This was a cross-sectional, quantitative, survey-based study. To maintain confidentiality, the identity of the participant remained anonymous throughout the entire study. The study was approved by the Institutional Review Board of Jordan University Hospital (67/2019/6219).

### Study population

Undergraduate university students were the main target population of the study. The university had approximately 35,000 students enrolled in 20 schools. Based on this figure, a 5% margin of error, a confidence of 95%, a 50% expected response rate, a minimum number of 380 responses were required. Inclusion criteria were: all undergraduate students of all academic schools at the University of Jordan, second-year and higher students, and students with any grade point average (GPA). Exclusion criteria were students not enrolled at the University of Jordan and first-year and graduate students. First-year students were excluded from the study since it was difficult to reach them considering that they were less organized in their first semester at the university.

### Study instrument

A recently developed and validated questionnaire related to knowledge, attitude, and practice towards biomedical research and biobanking among the public in Arab countries was used as a model of the questionnaire used in this study with some modifications [10]. A pilot study was first conducted on 40 university students of various disciplines for 3 weeks in October 2019 to provide insight and feedback regarding the suitability and clarity of the questions. Minor modifications of the questions were made accordingly. The final questionnaire comprised 5 sections. The first section included an introductory page that explained the purpose behind the questionnaire and included a note that the study was ethically approved, responses would remain anonymous, and participation was voluntary. A question of whether they agreed to participate was provided to them with a yes/no option. Respondents were notified that participation would be regarded as their informed consent. The second section covered demographics data. The third section included questions regarding participating in biomedical research and the influence of certain factors on the decision to participate including the time needed to provide a biospecimen, type of biospecimen, direct health benefit from participating, religious point of view regarding participating, privacy options, withdrawal options, financial benefit from participating, and trust in researchers. The fourth section included biobanking concepts, biospecimen donation options, and the influence of certain biobanking-related issues. These included the type of future research done, direct benefit from the results, biobank-based institution re-contact conditions, level of informed consent, and how withdrawal may influence and change decisions. Survey respondents had the chance to add a comment at the end of the survey. The questionnaires, in both English and Arabic, used can be seen as Additional file 1 and Additional file 2, respectively.

### Data collection

The preliminary and final questionnaires were uploaded on Google Forms (http://docs.google.com/forms/). The weblinks of the questionnaires were distributed to students. The final version of the questionnaire was randomly distributed to students via Facebook, WhatsApp, Microsoft Teams, and electronic mail through the University Student Union, online student groups, and student representatives of the different schools and majors. Facebook and WhatsApp are the two most commonly used platforms by undergraduate students at the University of Jordan. Data collection lasted for two months in order to reach the largest possible number of responses, particularly students from non-health sciences schools who were difficult to reach.

### Measures

#### Demographic data

Participants were asked about their sex, age, school (all 20 schools were included as drop-down menu), academic year (second, third, and fourth and higher), GPA (below 2, 2.00–2.49, 2.50–2.99, 3.00–3.49 and 3.50–4.00), and monthly income of the family in Jordanian Dinar (< 500, 500–999, 1000–1499, 1500–2000, or 2000 and higher; 1 Jordanian Dinar = $1.41). The students were categorized according to their school of affiliation and comparisons were made as follows: medical versus non-medical, health versus non-health, and scientific versus non-scientific.

### Perceptions, attitudes, and practice towards biospecimens-based research

Initially, participants were asked if they had ever participated in biomedical research before, what they thought of donating biospecimens for research purposes, and their willingness to contribute. Those attitudes were measured on a 5-point Likert scale with scores ranging from ‘‘very unlikely’’ to ‘‘very likely’’ in addition to a category of ‘‘don’t know’’. Subsequently, the respondents were asked to specify the type of biospecimen(s) they would be willing to donate giving them several options (blood, cheek swabs, urine, saliva, stool, and tissue leftover). Moreover, participants were asked to indicate the importance of certain factors when donating a specimen like revealing personal and family-related information. Analyzed factors were: time spent in donating, fear of needles/blood, the existence of informed consent prior to participation, availability of direct medical benefit, religious views, confidentiality, ability to withdraw biospecimen and data, type of collected personal and family-based information, approval of an authorized ethics committee, type of research, the impact of the research on public health, availability of general or personalized results to the donor, and the identity of and the level of trust towards researchers.

### Perceptions and attitudes towards biobanking

The concept of a biobank was first defined to students as they were then asked if they found it familiar. Then, they were given nine statements measuring the extent of participants’ agreement on participating in a biobank. This included the overall possibility of participating in a biobank in general or the possibility of participating under certain situations. Such situations included the lack of benefit, the unavailability of general research results to the donor, undisclosed type of research, the nature of the managing body of the biobank (academic institution, governmental entity, private sector, an Arab entity, or a non-Arab entity).

Participants were also asked about their preferences regarding the level of informed consent, the type of biospecimen coding, the possibility of re-contact, and the fate of biospecimen and information upon withdrawal. They were given three options of informed consent: broad consent allowing researchers to conduct any type of research on the biospecimen, research-specific consent such as research on specific diseases, or one-time consent that necessitates contacting donors every time a biospecimen would be used for research.

### Data analysis

Data were analyzed using the SPSS software program, version 22.0 (SPSS Inc., Chicago, IL, USA). Descriptive statistics were used to report biospecimen characteristics in addition to frequencies and percentages. Pearson Chi-square was used to assess the relationship between individual responses within each statement with demographics. The correlations between responses within each statement and the overall willingness to participate in biobanking were also assessed. *P* values less than 0.05 were considered significant. Missing responses were not considered in the analysis.

## Results

The questionnaire reached 476 students, 435 of them consented to participate and 41 declined. Table 1 shows the sociodemographic characteristics of the study sample. The majority of respondents were females (72.0%). The age range of the students was from 17 to 39 with a mean of 20.8. The distribution of student respondents according to schools was 45.5% from the School of Medicine, 73.1% from health schools, and 83.4% from scientific schools. The majority of responses (42.5%) belonged to students in the fourth year and higher and the lowest group constituted third-year students (22.1%). Over 90% of students had a GPA of 2.5 and higher. Those with GPA of 2.0–2.5 constituted 9.0% and failing students with GPA below 2.0 were a minority (0.7%). Students with variable family income were well represented with the highest proportions was those with income of 500–999 JD (23.4%) followed by 2000 JD (20.9%) and above and the lowest proportion was students with income of less than 500 JD (8.7%) and 1500–1999 JD (9.2%). All questions were answered by all student respondents except for 98 students who did not know or did not want to reveal their family income and one missing response for GPA.

**Table 1:** Sample characteristics

Sex (N = 435)	%
Male (122)	28.0
Female (313)	72.0
Age	
17 (1)	0.2
18 (11)	2.5
19 (124)	28.5
20 (89)	20.5
21 (81)	18.6
22 (49)	11.3
23 (54)	12.4
24 and above (26)	2.1
Mean = 20.8 (± 2.01; Min = 17; Max = 39)	
School categories	
Medical (198)	45.5
Non-medical (237)	54.5
Health School (318)	73.1
Non-health (117)	26.1
Scientific (363)	83.4
Non-scientific (72)	16.6
Academic years	
Second year (154)	35.4
Third year (96)	22.1
Fourth-year and higher (185)	42.5
GPA	
< 2.00 (3)	0.7
2.00–2.49 (39)	9.0
2.50–2.99 (106)	24.4
3.00–3.49 (144)	33.2
3.5–4.00 (142)	32.7
Income (JD)^*^	
< 500 (38)	8.7
500–999 (105)	23.4
1000–1499 (71)	15.2
1500–1999 (40)	9.2
2000 and higher (92)r	20.9

^*^Missing data (n) = 98 (22.5%)

### Knowledge, attitude, and practice towards biomedical research and biobanking

Students were asked if they had previously participated in biomedical research and over a quarter of them (28.9%) indicated that they did (Table 2). Previous participation in research was found to be significantly high among students of the medical, health, and scientific schools (*p* < 0.001). Students of higher academic years and those with higher income also had a significant tendency (*P* < 0.001 and 0.01, respectively) to previously participating in biomedical research. As for GPA, students with intermediate GPA, i.e. 2.50–2.99, were the largest group to previously participate in research (*P* < 0.01).

**Table 2:** Knowledge of biobanking, attitudes towards the use of biospecimen in research, and engagement in research

Questions	Responses		Significant factors associated with favorable response^a^
	Yes (%)	No (%)	
Have you previously participated in medical research	112 (28.9)	276 (71.1)	Being in medical^***^, health^***^, or scientific^**^ schools, 3rd and 4th year, GPA of 2.50–2.99^***^, income > 2000 JD^**^
Have you previously heard of biobanks?	230 (52.9)	250 (47.1)	Being in medical^***^, health^***^, or scientific^**^ schools, GPA of ≥ 2.50^*^, income of 500–1500 and > 2000 JD^**^, prior participation in biomedical research^*^

	Strongly agree (%)	Agree (%)	Disagree (%)	Strongly disagree (%)	
Do you generally agree with using biospecimen in medical research?	169 (38.9)	250 (57.5)	16 (3.7)	0 (0)	Being in medical^***^, health^***^, or scientific^***^ schools, GPA of ≥ 3.50^*^, income 500–1500 and > 2000 JD^**^, prior participation in biomedical research^**^
How likely is it that you participate in biomedical research by providing biospecimen and personal/family information?	145 (35)	246 (59.4)	20 (4.8)	3 (0.7)	Being in medical^*^, health^**^, or scientific^*^ schools, prior participation in biomedical research^*^
How likely is it that you participate in genetic research?	153 (36.9)	213 (51.3)	39 (9.4)	10 (2.4)	Being in medical^*^, health^**^, or scientific^*^ schools, income 500–1500 and > 2000 JD^**^
Possibility of participating in future biobanking research	99 (24.6)	271 (67.4)	25 (6.2)	7 (1.7)	Being in medical^***^, health^*^, or scientific^*^ schools, income 500–1500 and > 2000 JD^*^, prior participation in biomedical research^**^

^a^*P < 0.05; **P < 0.01; ***P < 0.001)

Students were then asked if they agreed on the use of biospecimens in biomedical research. The majority either agreed (57.5%) or strongly agreed (38.9%) (Table 2). Students in medical, health, and scientific schools significantly tended to strongly agree compared to other schools (*P* < 0.001). As for income, those with an income of < 500 JD or 1500–1999 JD tended to be less accepting of the use of biospecimens in research (*P* = 0.023).

Students were then asked about their willingness to participate in biomedical or genetic research (Table 2). There is an overwhelming willingness to participate in both types of research where approximately 95% of students were willing to participate in biomedical research in general and almost 90% of them were willing to participate in genetic research. Willingness to participate in such research was not associated with sex, academic year, or GPA. Students from medical, health, and scientific schools were significantly more willing to participate in both compared to their counterparts. There is also a trend that students with a family income of < 500 JD or 1500–1999 JD were found to be less enthusiastic towards participation.

Students were then asked if they had heard of biobanks before (Table 2). More than half of them indicated that they heard of the term with significant association with being in medical, health, or scientific schools, having a GPA of more than 2.50, having a family income of 500–1500 or > 2000 JD, and prior participation in biomedical research. The majority of students (~ 90%) either strongly agreed or agreed to participate in biobanking (Table 2). Students of health schools were significantly more enthusiastic about participation in a biobank.

### Preferred biospecimen donation for biomedical research

**Fig. 1:**
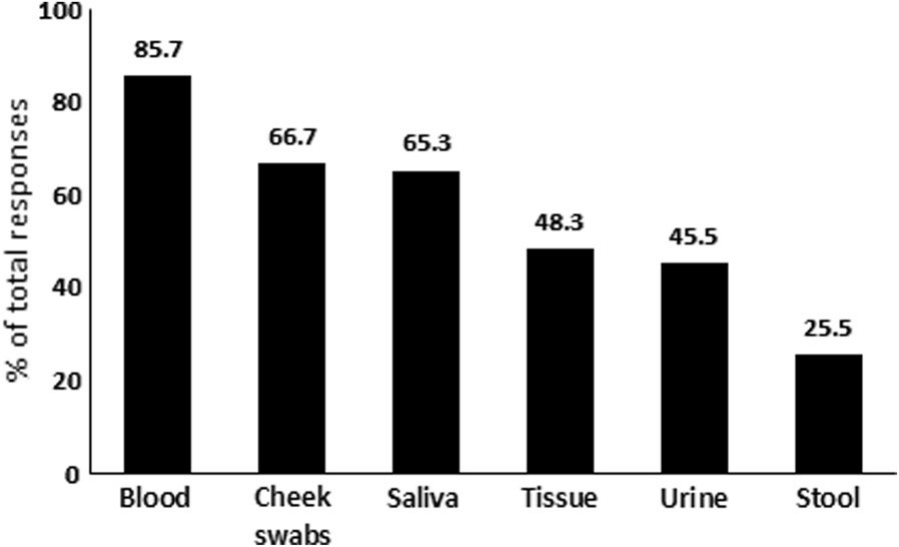
Preferences of biospecimens donated for research

Students were asked about the biospecimens they would prefer to contribute for research purposes. The most preferred biospecimen was blood (85.7%), followed by comparably cheek swabs (66.7%) and saliva (65.3%) (Fig. 1). On the other hand, stool (25.5%) was the least preferred biospecimen to be donated for research.

### Factors influencing participation in biomedical research and preferred biospecimen types

Questions regarding factors that might affect participation in biomedical research were also asked. As detailed in Table 3, the most influential factors that were ranked as “very important” were protection of privacy (55.9%), followed closely by consenting prior to participation (54.3%), approval of an authorized ethics committee (51.5%), and trust towards researchers (45.5%). On the other hand, the least important factors were personal gain of any profit (66.7%), fear of blood and needles and (65.3%), and identity of researchers (43.4%). Interestingly, religious views and time spent to participate were considered to be “important” by a considerable proportion of students (44.6%).

**Table 3:** Factors influencing participation in biomedical research

	Least level of importance (%)	Slightly important (%)	Important (%)	Very important (%)	Unsure (%)
Time spent to participate	23 (5.3)	127 (29.2)	194 (44.6)^*^	69 (15.9)	22 (5.1)
Fear of blood and needles	**284 (65.3)**	63 (14.5)	50 (11.5)	19 (4.4)	19 (4.4)
Consenting prior to participation	12 (2.8)	33 (7.6)	148 (34.0)	**236 (54.3)**	6 (1.4)
Gaining direct personal benefit	**137 (31.5)**	95 (21.8)	107 (24.6)	59 (13.6)	37 (8.5)
Religious views on participation	23 (5.3)	127 (29.2)	**194 (44.6)**	69 (15.9)	22 (5.1)
Protection of privacy	16 (3.7)	38 (8.7)	131 (30.1)	**243 (55.9)**	7 (1.60
Ability of withdrawal	67 (15.4)	**107 (24.6)**	**121 (27.8)**	**108 (24.8)**	32 (7.4)
Nature of provided information	35 (8.0)	86 (19.8)	**153 (35.2)**	**145 (33.3)**	16 (3.7)
Nature of family information provided	33 (7.6)	90 (20.7)	**148 (34.0)**	**150 (34.5)**	14 (3.2)
Approval of an authorized ethics committee	19 (4.4)	56 (12.9)	132 (30.3)	**224 (51.5)**	4 (0.9)
Type of research conducted	64 (14.7)	98 (22.5)	**138 (31.7)**	120 (27.6)	15 (3.4)
Positive impact on public health	9 (2.1)	75 (17.2)	**170 (39.1)**	**169 (38.9)**	12 (2.8)
Personal gain of any profit	**290 (66.7)**	70 (16.1)	21 (4.8)	7 (1.6)	47 (10.8)
Availability of general research results	26 (6.0)	101 (23.2)	**171 (39.3)**	130 (29.9)	7 (1.6)
Availability of personalized research results	27 (6.2)	84 (19.3)	**170 (39.1)**	142 (32.6)	12 (2.8)
Trust towards researchers	11 (2.5)	61 (14.0)	153 (35.2)	**198 (45.5)**	12 (2.8)
Identity of researchers	**189 (43.4)**	85 (19.5)	74 (17.0)	39 (9.0)	48 (11.0)

^*^Prominent responses are bolded

### Influence of biobanking aspects on willingness to donate

To investigate how the impact of certain aspects related to biobanks would affect the willingness to participate in a biobank, students were asked if they were willing to donate for a biobank under certain situations. The likeliness of students to participate in biobanking drastically dropped from 92.0% to less than 30% when they were told that they would not know the type of research conducted on their biospecimen. The unavailability of general research results to the donor also reduced the likeliness to participate to less than 50%. However, lack of personal benefits had a less impact where 81.1% indicated that they would still participate. In addition, knowing that the biobank would be managed by an academic institution had a favorable influence compared to a governmental entity, a private sector, an Arab institution, or a non-Arab institution (89.2%, 75.9%, 63.4%, 76.3 and 66.9, respectively) (Table 4).

**Table 4:** Impact of certain aspects of biobanks on willingness to participate

Possibility of participating in a biobank under different circumstances	Strongly disagree (%)	Disagree (%)	Agree (%)	Strongly agree (%)	Unsure (%)
Overall likelihood to participate	7 (1.7)	25 (6.2)	271 (67.4)	99 (24.6)	33 (7.5)
a. Lack of benefits	13 (3.0)	36 (8.3)	242 (55.6)	111 (25.5)	33 (7.6)
b. Undisclosed type of research	85 (19.5)	161 (37.0)	105 (24.1)	20 (4.6)	46 (10.6)
c. Unavailability of general research results	42 (9.7)	138 (31.7)	175 (40.2)	34 (7.8)	46 (10.6)
d. If managed by a governmental center	19 (4.4)	39 (9.0)	250 (57.5)	80 (18.4)	47 (10.8)
e. If managed by an academic institute	8 (1.8)	22 (5.1)	237 (54.5)	151 (34.7)	17 (3.9)
f. If managed by a private health sector	32 (7.4)	66 (15.2)	208 (47.8)	68 (15.6)	61 (14)
g. If managed by an Arab institute	16 (3.7)	46 (10.6)	249 (57.2)	83 (19.1)	41 (9.4)
h. If managed by a non-Arab institute	26 (6.0)	63 (14.5)	217 (49.9)	74 (17.0)	55 (12.6)

### Personal choices regarding biobanking aspects

Students were then asked about their preferences in specific biobank operations. As shown in Table 5, there was no clear preference over which type of informed consent was preferred by students where approximately one-third of respondents chose any one of the three types. In addition, half of the students preferred biospecimens to be traceable back to donors, one quarter of them either preferred de-identifying biospecimens irreversibly either at the time of collection or upon request at a later stage.

**Table 5:** Specific preferences in regards to biobanking-related aspects

*Preferred level of informed consent when participating in a biobank and donating a biospecimen*	
Broad consent	138 (31.7)
Research-specific consent	166 (38.2)
One-time consent with conditional re-contact	131 (30.1)
*Preferred procedure to protect donor identity when collecting biospecimens*	
Code biospecimens with possible re-identification	216 (49.7)
Irreversibly de-identify biospecimen at a later stage upon request	120 (27.6)
Irreversibly de-identify biospecimens at the time of collection	98 (22.5)
Neutral	1 (0.2)
*When to contact participants if specific results to their biospecimens are generated*	
Re-contact under all circumstances	293 (67.4)
Re-contact me only in definite cases of having or increasing the possibility of having a disease	100 (23.0)
Re-contact me only in definite cases of having or increasing the possibility of having a treatable disease	37 (8.5)
Never re-contact me	5 (1.1)
*In case of deciding to withdraw from a biobank*	
Disposal of biospecimens only	31 (7.1)
Deleting all data only	41 (9.4)
Disposal of both biospecimen and data	225 (51.7)
Removing donor identifiers, but keep biospecimens and data for future research	138 (31.7)

As for re-contacting donors, two-thirds of students opted to be re-contacted whenever results specific to their biospecimens would be generated. On the other hand, a quarter of them preferred to be re-contacted whenever results indicated the possibility of disease diagnosis, 8.5% wanted to be re-contacted if the disease could be treated, and only 1.1% never wanted to be re-contacted (Table 5).

Upon withdrawal, slightly more than half of the students preferred to have their biospecimens and data to be completely disposed of; however, one-third of them would choose irreversible elimination of donor identity but preserving biospecimens and related information (Table 5).

## Discussion

The term “biobank” appears to be new to almost half of student respondents from all disciplines whereas only 52.9% of the students stated that they have previous knowledge of the term. This figure is better than the 27% of Saudi healthcare students [11], the 40% of Egyptian medical students [12], and the 20% of Russian students [13] who knew what the term meant. However, it is less than the 83.7% of students of the Italian Padua University who were able to select the right definition from a multiple-choice question [14]. It is expected that students of medical, health, and scientific schools would know the term better compared with students of other disciplines. Knowledge is also correlated with previous participation in biomedical research signifying the actual participation in research to increase knowledge and improve attitude towards research.

In our study, an overwhelming majority of students expressed their willingness to participate in biomedical research (94.2%) and biobanking (92%). A high proportion of students (88.2%) were also willing to participate in genetic research. This enthusiasm to participate in a variety of biomedical research activities contrasts the attitude of Egyptian medical students who were by far less eager to engage in research [12]. Saudi healthcare students were similarly eager to donate for biobanks in association with prior tissue donation and testing, but not involved in medical research [11]. However, this study shows that willingness to participate was affected by prior participation in biomedical research as well as by school affiliation where students from health schools showed greater enthusiasm. Previous participation in biomedical research was also found to correlate with willingness to donate for biobanking among Egyptian students [12]. The sociodemographic status also did not influence students’ willingness to participate in biobanking among university students in Russia [13]. On the other hand, the survey conducted among students in Italy showed that sex has a significant association with participation [14]. A previous study among the Jordanian population showed that positive responses correlated significantly with younger age and increasing education, but not sex [8].

Besides enthusiasm in participating in biobanks observed among this young generation, two major themes are found to play a major role in determining the likelihood of participation. The first is the great value of personal autonomy. This can be revealed by student responses to statements related to the protection of privacy, the ability to withdraw data, the type of information provided to researchers including family information. Confidentiality could also be noticed by student preferences at the time of donation and in case of withdrawal from biobanks. Half of the students preferred to be able to remove personal identifiers of biospecimens either at a later stage of donation or at the time of donation. In addition, about half of students preferred disposal of both biospecimens and data and 30% would rather biobanks keep biospecimens but de-identify donors upon withdrawal. Concerns about confidentiality were one of the main reasons Saudi and Egyptian students were unwilling to donate to biobanks [11, 12].

The other theme that influences student participation in biobanks is the type of research. A large number of students indicated that it was important for them to know what kind of research would be conducted on their biospecimens and this was confirmed by the shift in willingness to participate in biobanking if they do not know this information. The study surveying Russian students [13] showed that the specific goal of the research had the greatest impact on one’s decision to participate. Saudi and Egyptian students also expressed their concern of misuse of their biospecimens to be a major determining factor to their participation [11, 12].

In addition, although students indicated that the type of researchers handling the biospecimens was not important, willingness to donate for a biobank is influenced according to the entity operating this biobank with an academic institution having the most trust and non-Arab and private biobanking having the least. This trend appears to be a common phenomenon as has been illustrated by two reviews of the literature on this topic covering different nations such as the U.S., Scotland, China, among many others [15, 16]. This attitude is not restricted to the general public, but also to healthcare professionals who expressed willingness to participate in biobanks affiliated with a hospital, university, or government research institutions and less likely with for-profit, commercial establishments [17].

Importantly, fear of blood and needles was not a major factor in preventing students from participating in biomedical research, in addition, blood was selected as the most preferred biospecimen to donate followed by cheek swabs and saliva. Stool, on the other hand, was the least preferred. Similarly, fear of needles was one of the stated factors that would prevent Saudi and Egyptian students from participating in biobanks [11, 12], although blood and saliva were the most preferred biospecimens to be donated. Blood appears to be an acceptable biospecimen to be donated by the public in Egypt [18] as well as others [19, 20].

The informed consent of research participants is a cornerstone of biomedical research. A majority of students (88.4%) believe consenting before participation is an important factor for them to participate. However, there was no consensus on the preferred type of informed consent with selection almost equally distributed among three options: broad consent, research-specific consent, or one-time consent with possible renewal. In a previous population-based study conducted in Jordan, the majority of respondents (75%) favored broad consent and only 4% preferred re-consenting for every study [21]. This was contrary to the findings of another study that reported that only half of the patients with multiple sclerosis preferred broad consent and 37% would prefer to be re-consented [22]. It is tempting to argue that the high preference for re-consenting and re-contacting is due to the eagerness to learn of the type of research to be conducted on participants’ biospecimens. This argument is based on the overwhelming strong support for research in Jordan [21]. However, mistrust in biobanks and/or researchers cannot be overlooked. It is, therefore, of interest to investigate if the choice of re-consenting and re-contacting is due to interest in research, mistrust, or another factor.

There has been considerable discussion about the issue of returning research results for some time now and guidelines are needed to be established taking into consideration the type of data to be returned, donor’s rights, and the elaborate logistics associated with this process [23]. Returning of research results has been found to strongly correlate with willingness to participate in biobanking initiatives [24]. In this study, a favorable association between returning research results with re-consenting and re-contacting also appears to be a strong incentive for students to participate in biobanking. More than two-thirds of students want to be re-contacted under all circumstances when research results are generated. In addition, one-third of them want to be re-contacted only if the results reveal a certain disease. Elucidation of the type of research results of interest to students is another area to be investigated further.

In addition to sharing biospecimens, biobanks are associated with data sharing. The issue of data sharing, particularly, genomic data has been under investigation and debate for some time. Regulations such as the Genomic Data Sharing (GDS) policy in the US [25] and the General Data Protection Regulation (GDPR) to biobanking in Europe [26] have already been set up. There have also been calls to revisit regulations related to data sharing in two high-income countries, namely Canada and Australia [27, 28]. A disparate rate of approval among students at the University of Jordan to participate in biobanking was noted depending on the identity of researchers. Students were less eager to participate if the researchers are identified as non-Jordanian and, particularly, non-Arab. This is not restricted to Jordanian students where Egyptians also expressed concern about the export of their biospecimens abroad [29]. The same is also true in the US where survey respondents were less in favor of having their biospecimens and data be shared with researchers outside the US [30]. In two multi-national studies, the identity of researchers was a significant and common factor in determining public acceptance of data sharing [31, 32]. Hence, transparency of biobanks and researchers in dealing with biospecimens and data, in addition to the setting up of national guidelines for biospecimen and data sharing is necessary in order to increase trust among the public including university students, and to facilitate international collaboration.

## Conclusions

The results presented herein offer a promising outlook for biomedical research and biobanking in Jordan illustrating high student interest in these initiatives. Certain issues must be considered when attempting to involve the young generation in Jordan in biomedical research and biobanking. These issues include clarity regarding the concepts, purposes, and operational procedures of biobanks as well as the outcome of research from a personalized perspective. However, further and in-depth investigation regarding particular aspects related to biobanking is needed including data sharing, level of participation, and return of research results.

A major strength of this study is the inclusion of students of different disciplines with various demographic backgrounds representing a diverse group and, hence, providing credibility to the generated data. However, some limitations are worth noting. First, responses were based on a self-reporting, electronic questionnaire, a method that could overestimate students’ understanding of some items or may allow them to answer the questionnaire hastily. Additionally, using online data collection platforms could have prevented reaching a certain segment of students, i.e. those with lower academic standing (i.e. GPA) or from non-scientific disciplines.

## Supplementary Information

**Additional file 1**. Survey (in English)

**Additional file 2**. Survey (in Arabic)

## Data Availability

The datasets during and/or analyzed during the current study are available from the corresponding author on reasonable request.

## Abbreviation

GPA: Grade-point average

## References

[1] De Oliveira L, Dias MAB, Jeyabalan A, Payne B, Redman CW, Magee L, et al. Creating biobanks in low and middle-income countries to improve knowledge—the PREPARE initiative. In: Pregnancy Hypertension. 2018. p. 62–4. 10.1016/j.preghy.2018.05.007 PMC6134341 30177073

[2] Klingstrom T, Mendy M, Meunier D, Berger A, Reichel J, Christoffels A, et al. Supporting the development of biobanks in low and medium income countries. In: 2016 IST-Africa Conference, IST-Africa 2016; 2016. 10.1109/ISTAFRICA.2016.7530672

[3] Ahram M, Soubani M, Abu Salem L, Saker H, Ahmad M (2015). Knowledge, attitudes, and practice regarding genetic testing and genetic counselors in Jordan: a population-based survey. J Genet Couns.

[4] Coppola L, Cianflone A, Grimaldi AM, Incoronato M, Bevilacqua P, Messina F, et al. Biobanking in health care: evolution and future directions. J Transl Med. 2019; 172. 10.1186/s12967-019-1922-3 PMC6532145 31118074

[5] De Souza YG, Greenspan JS (2013). Biobanking past, present and future: responsibilities and benefits. AIDS.

[6] Vaught J (2020). Biobanking during the COVID-19 pandemic. Biopreserv Biobank.

[7] Hawkins A (2010). Biobanks: importance, implications and opportunities for genetic counselors. J Genet Couns.

[8] Ahram M, Othman A, Shahrouri M (2012). Public perception towards biobanking in Jordan. Biopreserv Biobank.

[9] Bossert S, Kahrass H, Strech D (2018). The public’s awareness of and attitude toward research biobanks: a regional German survey. Front Genet.

[10] Abd ElHafeez S, Ahram M, Abdelhafiz AS (2021). Development and validation of a biobank questionnaire intended for the public in the Arab region. Biopreserv Biobank.

[11] Merdad L, Aldakhil L, Gadi R, Assidi M, Saddick SY, Abuzenadah A (2017). Assessment of knowledge about biobanking among healthcare students and their willingness to donate biospecimens. BMC Med Ethics.

[12] Ziady H, El Zeiny N, Sultan E, El Sharef Y (2017). Assessment of medical students’ knowledge and attitude towards biobanks and biospecimens donation. J Med Res Inst.

[13] Tsvetkova LA, Eritsyan KY, Antonova NA (2016). Russian students’ awareness of and attitudes toward donating to biobanks. Psychol Russ State Art.

[14] Tozzo P, Fassina A, Caenazzo L (2017). Young people’s awareness on biobanking and DNA profiling: results of a questionnaire administered to Italian university students. Life Sci Soc Policy.

[15] Garrison NA, Sathe NA, Antommaria AHM, Holm IA, Sanderson SC, Smith ME (2016). A systematic literature review of individuals’ perspectives on broad consent and data sharing in the United States. Genet Med.

[16] Domaradzki J, Pawlikowski J (2019). Public attitudes toward biobanking of human biological material for research purposes: a literature review. Int J Environ Res Public Health.

[17] Caixeiro NJ, Byun HL, Descallar J, Levesque JV, De Souza P, Soon Lee C (2016). Health professionals’ opinions on supporting a cancer biobank: Identification of barriers to combat biobanking pitfalls. Eur J Hum Genet..

[18] Abdelhafiz AS, Sultan EA, Ziady HH, Ahmed E, Khairy WA, Sayed DM (2019). What Egyptians think. Knowledge, attitude, and opinions of Egyptian patients towards biobanking issues. BMC Med Ethics.

[19] Lewis C, Clotworthy M, Hilton S, Magee C, Robertson MJ, Stubbins LJ (2013). Public views on the donation and use of human biological samples in biomedical research: a mixed methods study. BMJ Open..

[20] Vaz M, Vaz M, Srinivasan K (2015). Listening to the voices of the general public in India on biomedical research—an exploratory study. Indian J Med Ethics.

[21] Ahram M, Othman A, Shahrouri M (2013). Public support and consent preference for biomedical research and biobanking in Jordan. Eur J Hum Genet.

[22] Ahram M, Zaza R, Ibayyan L, Dahbour S, Bahou Y, El-Omar A (2014). Towards establishing a multiple sclerosis biobank in Jordan. Int J Neurosci.

[23] Bledsoe MJ (2017). Ethical legal and social issues of biobanking: past, present, and future. Biopreserv Biobank.

[24] Ahram M, Othman A, Shahrouri M, Mustafa E (2014). Factors influencing public participation in biobanking. Eur J Hum Genet.

[25] Office of the Federal Register, National Archives and Records Administration. (2014, August 28). *79 FR 51345—Final NIH Genomic Data Sharing Policy*. [Government]. Office of the Federal Register, National Archives and Records Administration

[26] Regulation, C. (2016). 679 of 27 April 2016 on the protection of natural persons with regard to the processing of personal data and on the free movement of such data, and repealing Directive 95/46/EC. OJ L119/1

[27] Eckstein L, Chalmers D, Critchley C, Jeanneret R, McWhirter R, Nielsen J, Otlowski M, Nicol D (2018). Australia: regulating genomic data sharing to promote public trust. Hum Genet.

[28] Thorogood A (2018). Canada: will privacy rules continue to favour open science?. Hum Genet.

[29] Abou-Zeid A, Silverman H, Shehata M, Shams M, Elshabrawy M, Hifnawy T, Rahman SA, Galal B, Sleem H, Mikhail N, Moharram N (2010). Collection, storage and use of blood samples for future research: views of Egyptian patients expressed in a cross-sectional survey. J Med Ethics.

[30] Majumder MA, Cook-Deegan R, McGuire AL (2016). Beyond our borders? Public resistance to global genomic data sharing. PLOS Biol.

[31] Middleton A, Milne R, Almarri MA, Anwer S, Atutornu J, Baranova EE, Bevan P (2020). Global public perceptions of genomic data sharing: What shapes the willingness to donate DNA and health data?. Am J Hum Genet.

[32] Milne R, Morley KI, Almarri MA (2021). Demonstrating trustworthiness when collecting and sharing genomic data: public views across 22 countries. Genome Med.

