# Supplementary material for: Views of university students in Jordan towards Biobanking
Source: BMC Med Ethics. 2021 Nov 13;22:152. doi: 10.1186/s12910-021-00719-y (PMC8590123; doi:10.1186/s12910-021-00719-y)
Supplement: Supplementary file 1 — Additional file 1. Survey (in English) [file 12910_2021_719_MOESM1_ESM.pdf]

Views of University Students in Jordan toward Biobanking

This questionnaire aims to assess students' perception and attitudes toward, and willingness to participate in biomedical research via donating biological samples and storing them in a biobank for research purposes. The Institutional Review Board of Jordan University Hospital approved the survey. In case you agree to participate, please be sure that the collected information will remain confidential and will be used for research purposes only. Your identity will not be revealed. It will take 10-15 minutes to answer the questions.

1. I Agree to fill out this questionnaire:

   ☐ Yes (Move to next section)
   ☐ No (terminate)

Please answer the following questions in the best way you could

A. Personal Background Information:

2. Sex :

   ☐ Male
   ☐ Female

3. What is your age (in years)? -------------

4. Type of the school you study in:

   ☐ School of Medicine
   ☐ School of Dentistry
   ☐ School of Nursing
   ☐ School of Pharmacy
   ☐ School of Rehabilitation science
   ☐ School of Science
   ☐ School of Agriculture
   ☐ School of Engineering
   ☐ School of Information Technology (IT)
   ☐ School of Art
   ☐ School of Shari'a (Islamic Jurisprudence)
   ☐ School of Educational Science
   ☐ School of Law
   ☐ School of Sport Science
   ☐ School of Art and Design
   ☐ School of Foreign Languages
   ☐ School of Archaeology and Tourism
   ☐ School of International Studies
   ☐ Other

5. Year of university study:

- ☐ First
- ☐ Second
- ☐ Third
- ☐ Fourth or higher

6. Your grade point average (GPA):

- ☐ Less than 2.00
- ☐ 2.00-2.49
- ☐ 2.50-2.99
- ☐ 3.00-3.49
- ☐ 3.50-4.00

7. Religion:

- ☐ Muslim
- ☐ Christian
- ☐ Others

8. Your father's educational level

- ☐ Sixth grade or less
- ☐ Seventh grade-high school
- ☐ Diploma or higher

9. Your mother's educational level

- ☐ Sixth grade or less
- ☐ Seventh grade-high school
- ☐ Diploma or higher

10. Family income per month :

- ☐ Less than 500 JD
- ☐ 500-999 JD
- ☐ 1000-1499 JD
- ☐ 1500- 1999 JD
- ☐ 2000 JD or more
- ☐ I do not know/ I do not want to say

B. Participation in a medical research

11. Have you ever participated in a medical research/study?

- ☐ Yes

- [ ] No
- [ ] I cannot remember

12. Do you agree in general to use biomedical samples (like urine, blood, tissue biopsy, and buccal swabs) in medical research?

    - [ ] Strongly agree
    - [ ] Agree
    - [ ] Disagree
    - [ ] Strongly disagree

13. In general, what is the possibility that you participate in medical research via donating a biological sample (urine, blood, tissue biopsy) in addition to your and/or your family's personal or medical information?

    - [ ] Highly likely
    - [ ] Likely
    - [ ] Unlikely
    - [ ] Highlly unlikely
    - [ ] I am not sure

14. What is the possibility to participate in genetic research?

    - [ ] Highly likely
    - [ ] Likely
    - [ ] Unlikely
    - [ ] Highlly unlikely
    - [ ] I am not sure

15. Regarding the factors that determine the possibility of your participation, sample donation, and sharing your medical information in any medical research, please indicate the degree of importance for each of the following factors:

	Least Important	Somewhat Important	Important	Very important	I am not sure
The needed duration to donate a sample					
Fear of needles, if it is a blood sample					
The need for my permission before donation					
Getting a medical benefit in exchange for my donation					
My religion's opinion regarding samples donation					
The measures to protect my confidentiality by researchers					

The possibility of withdrawing my samples and information anytime I want					
Type of personal and medical information I will provide to researchers					
Type of personal and medical information regarding my family provided to the researchers					
Approval of an official ethics committee of the research that will use my samples or information.					
The type of research that will use my information and samples (such as genetic research, drug study,…etc)					
The positive influence of the research on community health					
Getting a financial benefit from the research					
The possibility of knowing the final general research results performed on my samples					
The possibility of receiving personalized research results regarding my samples					
The degree of my trust for the researchers to protect my samples and information					
The identity of the researchers (such as Jordanians, Arabs, non-Arab,.. etc)					

16. If you participated in medical research, which of the following samples do you agree to participate with? (you can choose more than one option)

    ☐ I do not wish to participate in medical research in the future
    ☐ Blood sample
    ☐ Buccal swab
    ☐ Urine sample
    ☐ Saliva
    ☐ Stool sample
    ☐ Tissue sample (left from a previous diagnostic, therapeutic, or surgical procedure)

Biobanks are medical research facilities that store various biological samples donated by healthy individuals or patients in addition to their personal, health, and sometimes family data, and data about the samples they donated, in order for researchers to use these samples and stored data In multiple future research to find new diagnostic or therapeutic means for various diseases, especially incurable diseases such as cancer. These research works are approved by official institutional research ethics committees (for example: the Scientific Research Ethics Committee at Jordan University Hospital).

17. Have you ever heard about the term "biobank"?

- ☐ Yes
- ☐ No

18. Attitudes toward participation and sample donation for a biobank

	Strongly disagree	Disagree	Agree	Totally agree	I am not sure
I believe it is possible that I give a biological sample for a biobank to be used in research in the future					
I believe it is possible that I give a biological sample for a biobank even if I do not know what type of research will be performed on it					
I believe it is possible that I give a biological sample for a biobank even if I will not be provided with any benefits					
I believe it is possible that I give a sample for a biobank even if I will not be provided with general research results					
I believe it is possible that I give a biological sample for a biobank managed bya governmental health center					
I believe it is possible that I give a biological sample for a biobank mnaged by an academic institute such as Jordan University Hospital					
I believe it is possible that I give a biological sample for a biobank managed by a private health center or a pharmaceutical company					
I believe it is possible that I give a sample for a biobank managed by an Arab institute					
I believe it is possible that I give a sample for a biobank managed by a Non-Arab institute					

19. There might be several levels of consent on donating a biological sample to a biobank. Which level of consent you would feel select with? (choose one answer only)

☐ Broad consent, which allows the biobank to use the biological samples and information in any type of research in future and at any time.
☐Specific consent, which allows the biobank to use the biological samples and information in a specific type of research or a specific disease.
☐ None of the above. Participants must be contacted to get their approval for any research in the future.

20. When participating in a biobank via donating a biological sample, the samples are coded to obscure the identity of the donor. Which of the following is most acceptable to you to protect your privacy ? (choose one answer only)
☐Using the code with the possibility of revealing the identity of the participant in future.
☐The choice of delete coding later on in a way that prevents retrieving the information, upon donor request
☐Concealing the identity and the related information of the donor immediately after sample donation

21. When should you be contacted as a donor regarding your samples' results? (choose one answer only)

☐ In all cases
☐ Only in case the results show a confirmed diagnosis of a disease or an increased possibility of developing a disease
☐ Only in case the results show a confirmed disease or an increased possibility of a certain disease that can be treated
☐ I should not be recontacted in any case

22. In case you decide to withdraw your participation in a biobank, which of the following, in your opinion, should lead to withdrawal of consent? (choose one answer only)
☐Sample disposal only
☐Deletion of all information only
☐Sample disposal and deletion of all information
☐Deletion of the identity of the donor and the identifying data completely with the possibility of using the sample and data in a new research

Thank you for your participation. You can leave your comments below if you have any.
