# Supplementary material for: Views of university students in Jordan towards Biobanking
Source: BMC Med Ethics. 2021 Nov 13;22:152. doi: 10.1186/s12910-021-00719-y (PMC8590123; doi:10.1186/s12910-021-00719-y)
Supplement: Supplementary file 2 — Additional file 2. Survey (in Arabic) [file 12910_2021_719_MOESM2_ESM.pdf]

# أفكار الطلبة ومواقفهم وسلوكياتهم المحتملة حيال التبرع بعينات حيوية بشرية وتخزينها في بنك حيوي لأغراض بحثية طبية

يهدف هذا البحث إلى معرفة أفكار الأفراد من فئة طلاب الجامعة الأردنية ومواقفهم حيال المشاركة في أبحاث طبية والتبرع بعينات حيوية وتخزينها لدى بنوك حيوية لأغراض بحثية، وفي حال موافقتك للمشاركة إلى هذه الدراسة الاستقصائية، فتأكد أن البيانات المجمعة ستكون سرية وتستخدم لأغراض علمية فقط. يستغرق الاستبيان من 5 الى 10 دقائق . الرجاء تعبئة الاستبيان بكل صدق و شفافية حيث ستكون هويتك مجهولة تماما، علماً بأن لجنة أخلاقيات البحث العلمي في مستشفى الجامعة الأردنية قد وافقت على هذه الدراسة.

* Required

## Untitled Title

1. بناءً على ما سبق من الفقرة التعريفية, أوافق على ملىء هذا الاستبيان *

*Mark only one oval.*

- ( ) أوافق
- ( ) لا أوافق

*Skip to question 2*

## البيانات الشخصية الأساسية

يرجى الإجابة على الأسئلة التالية على أفضل نحو ممكن

2. الجنس *

*Mark only one oval.*

- ( ) ذكر
- ( ) أنثى

3. العمر ( بعدد السنوات ) *

______________________

4. الكلية *

*Mark only one oval.*

- الطب
- طب الأسنان
- التمريض
- صيدلة
- علوم التأهيل
- العلوم
- الزراعة
- الهندسة
- تكنولوجيا المعلومات
- الآداب
- الأعمال
- الشريعة
- علوم تربوية
- حقوق
- تربية رياضية
- الفنون و التصميم
- اللغات الأجنبية
- الآثار و السياحة
- الدراسات الدولية
- أخرى

5. السنة الدراسية الجامعية *

*Mark only one oval.*

- الأولى
- الثانية
- الثالثة
- الرابعة او أعلى

6. معدلك الدراسي

*Mark only one oval.*

- ( ) أقل من 2.00
- ( ) من 2.00 إلى 2.49
- ( ) من 2.50 إلى 2.99
- ( ) من 3.00 إلى 3.49
- ( ) من 3.50 إلى 4.00

7. الديانة *

*Mark only one oval.*

- ( ) الإسلام
- ( ) المسيحية
- ( ) أخرى

8. المستوى التعليمي للأب *

*Mark only one oval.*

- ( ) الصف السادس أو أقل
- ( ) من الإعدادي إلى الثانوية العامة
- ( ) دبلوم وأعلى

9. المستوى التعليمي الأم *

*Mark only one oval.*

- ( ) الصف السادس أو أقل
- ( ) من الإعدادي إلى الثانوية العامة
- ( ) دبلوم وأعلى

10. الدخل العائلي الشهري *

*Mark only one oval.*

- ( ) أقل من 500 دينار
- ( ) 500-999 دينار
- ( ) 1000-1499 دينار
- ( ) 1500-1999 دينار
- ( ) 2000 وما يزيد
- ( ) لا أعلم / لا أود الافصاح

## المشاركة في البحث الطبي

11. هل سبق أن شاركت في بحث طبي؟ *

*Mark only one oval.*

- ( ) نعم
- ( ) لا
- ( ) لا أذكر

12. هل توافق بشكل عام على استخدام العينات الحيوية (أمثلة: سوائل الجسم من دم وبول، أوعينات من الأنسجة الحيوية كالخزعات ومسحات من داخل الخد)، في الأبحاث الطبية؟ *

*Mark only one oval.*

- ( ) أوافق بشده
- ( ) أوافق
- ( ) أعارض
- ( ) أعارض بشدة

13. ما مدى احتمالية مشاركتك ببحث طبي من خلال تزويد الباحثين بعينة حيوية بالإضافة لمعلومات شخصية وصحية عنك و/أو عن عائلتك، بشكل عام؟ *

*Mark only one oval.*

- محتمل جدا
- محتمل
- غير محتمل
- غير محتمل أبدا
- غير متأكد

14. ما مدى احتمالية مشاركتك في بحث جيني/وراثي؟

*Mark only one oval.*

- محتمل جدا
- محتمل
- غير محتمل
- غير محتمل أبدا
- غير متأكد

15. فيما يخص قرار المشاركة في أي بحث طبي والتبرع بعينة حيوية وتقديم بيانات، يرجى تحديد درجة أهمية العوامل التالية في قرارك. *

*Mark only one oval per row.*

| | أهمية قصوى | هام | هام بعض الشيء | أدنى درجة في الأهمية | غير متأكد |
|---|---|---|---|---|---|
| الفترة الزمنية اللازمة للمشاركة والتبرع بعينة. | ⬭ | ⬭ | ⬭ | ⬭ | ⬭ |
| إذا كانت العينة الحيوية هي الدم، فأنا أخشى الإبر و/أو الدم | ⬭ | ⬭ | ⬭ | ⬭ | ⬭ |
| طلب موافقتي قبل للمشاركة. | ⬭ | ⬭ | ⬭ | ⬭ | ⬭ |
| حصولي على استفادة طبية مباشرة لصحتي من تبرعي | ⬭ | ⬭ | ⬭ | ⬭ | ⬭ |
| رأي معتقدي الديني فيما يتعلق بالتبرع بعينات حيوية | ⬭ | ⬭ | ⬭ | ⬭ | ⬭ |
| إجراءات حماية خصوصيتي لدى الباحثين. | ⬭ | ⬭ | ⬭ | ⬭ | ⬭ |
| إمكانية الانسحاب وسحب عيناتي وبياناتي الطبية في أي وقت من قبل الباحثين | ⬭ | ⬭ | ⬭ | ⬭ | ⬭ |
| طبيعة معلوماتي الشخصية والصحية التي سأزودها للباحثين | ⬭ | ⬭ | ⬭ | ⬭ | ⬭ |
| طبيعة المعلومات العائلية التي سأزودها للباحثين | ⬭ | ⬭ | ⬭ | ⬭ | ⬭ |
| مصادقة لجنة أخلاقيات بحث معتمدة على البحث الذي سيتضمن استخدام عيناتي الحيوية وبياناتي. | ⬭ | ⬭ | ⬭ | ⬭ | ⬭ |
| نوع البحث الذي سيجرى على عيناتي وبياناتي الطبية (مثل بحث جيني/وراثي، اختبار دوائي ... إلخ) | ⬭ | ⬭ | ⬭ | ⬭ | ⬭ |
| التأثير الإيجابي للبحث على صحة المجتمع | ⬭ | ⬭ | ⬭ | ⬭ | ⬭ |
| حصولي على أي أرباح مادية من نتائج الأبحاث | ⬭ | ⬭ | ⬭ | ⬭ | ⬭ |
| إذا كان بإمكاني الحصول على النتائج العامة للأبحاث التي ستجرى على عينتي | ⬭ | ⬭ | ⬭ | ⬭ | ⬭ |
| إذا كان بإمكاني الحصول على النتائج البحثية الخاصة بي | ⬭ | ⬭ | ⬭ | ⬭ | ⬭ |

| | | | | | |
|---|---|---|---|---|---|
| حجم ثقتي بالباحثين للحفاظ على عيناتي وبياناتي | ◯ | ◯ | ◯ | ◯ | ◯ |
| هوية الباحثين (مثال: أردنيين، عرب، غير عرب، الخ) | ◯ | ◯ | ◯ | ◯ | ◯ |

16. إن أردت أن تشارك في بحث طبي، أي من العينات التالية توافق أن تشارك بها في بحث طبي؟

*Check all that apply.*

- [ ] لا أرغب في المشاركة في بحث طبي في المستقبل
- [ ] عينة دم
- [ ] مسحة من الخد
- [ ] بول
- [ ] لعاب
- [ ] براز
- [ ] (عينة نسيجية (المتبقي من فحص تشخيصي أو علاجي أو جراحي

## البنك الحيوي

البنوك الحيوية: هي مرافق للأبحاث الطبية لتخزين مختلف العينات الحيوية والتي يتبرع بها أشخاص أصحاء أو مرضى بالإضافة إلى بياناتهم الشخصية والصحية، وأحيانًا العائلية، وبيانات تتعلق بالعينات التي تبرعوا بها، ليستخدم الباحثون تلك العينات والبيانات المخزنة في أبحاث مستقبلية متعددة من أجل إيجاد طرق تشخيصية جديدة أوعلاجية لمختلف الأمراض، لا سيما الأمراض المستعصية كالسرطان، (وهذه الأبحاث تعتمدها لجان أخلاقيات بحثية مؤسسية معتمدة (مثال: لجنة أخلاقيات البحث العلمي في مستشفى الجامعة الأردنية.

17. هل سبق وأن سمعت بمصطلح "بنك حيوي"؟ *

*Mark only one oval.*

◯ نعم

◯ لا

18. التوجه للمشاركة والتبرع بعينة حيوية لبنك حيوي *

*Mark only one oval per row.*

| | أرفض بشدة | أرفض | أوافق | أوافق بشدة | غير متأكد |
|---|---|---|---|---|---|
| أعتقد أنه من المحتمل أن أقدم عينة حيوية لبنك حيوي لاستخدامها في أبحاث مستقبلية | | | | | |
| أعتقد أنه من المحتمل أن أقدم عينة حيوية لبنك حيوي وإن لم أكن على علم بطبيعة البحث الذي سيجرى على عينتي. | | | | | |
| أعتقد أنه من المحتمل أن أقدم عينة حيوية لبنك حيوي وإن لم ينتج عن ذلك أي استفادة مباشرة لي. | | | | | |
| أعتقد أنه من المحتمل أن أقدم عينة حيوية لبنك حيوي وإن لم أحصل على نتائج عامة للبحث الذي ستشارك به عيناتي ومعلوماتي | | | | | |
| أعتقد أنه من المحتمل أن أقدم عينة حيوية لبنك حيوي تديره مؤسسة صحية حكومية | | | | | |
| أعتقد أنه من المحتمل أن أقدم عينة حيوية لبنك حيوي تديره مؤسسة أكاديمية كمستشفى الجامعة الأردنية | | | | | |
| أعتقد أنه من المحتمل أن أقدم عينة حيوية لبنك حيوي تديره مؤسسة صحية تجارية كمستشفى خاص أو شركة دوائية | | | | | |
| أعتقد أنه من المحتمل أن أقدم عينة حيوية لبنك حيوي تديره مؤسسة عربية | | | | | |
| أعتقد أنه من المحتمل أن أقدم عينة حيوية لبنك حيوي تديره مؤسسة غير عربية | | | | | |

19. قد تكون هناك عدة درجات من الموافقة التي من الممكن أن تختارها عند مشاركتك وتبرعك بعينة لبنك حيوي. أي من الدرجات التالية قد تختار؟ (خيار واحد فقط) *

*Mark only one oval.*

- الموافقة واسعة النطاق، التي تسمح باستخدام العينات الحيوية والبيانات ذات الصلة في بحث مستقبلي غير محدد من أي نوع وفي أي وقت
- الموافقة محدودة النطاق، التي تسمح باستخدام العينات الحيوية والبيانات ذات الصلة فقط التي تجرى على مرض معين أو نوع معين من الأبحاث
- لا شيء مما سبق، إذ يجب إعادة الاتصال بالمشاركين للحصول على موافقتي لأي بحث مستقبلي.
- Other: ____________________

20. عند المشاركة بعينة في بنك حيوي، يتم ترميز العينات لإخفاء هوية المتبرع، أي مما يلي الأكثر قبولًا لك لحماية خصوصيتك كمشارك؟ (خيار واحد فقط) *

*Mark only one oval.*

- استخدام الرمز مع إمكانية الوصول لهوية المشارك في المستقبل
- إمكانية حذف الرمز لاحقاً على نحو لا يمكن الرجوع عنه بناءً على طلب المشارك ذلك
- إخفاء هوية صاحب العينة والبيانات مباشرة عند تجميعها
- Other: ____________________

21. متى يجب الاتصال بك كمتبرع عند ظهور نتائج خاصة بعينتك؟ (اختر/ي إجابة واحدة فقط) *

*Mark only one oval.*

- في جميع الحالات
- فقط في الحالات المؤكدة للإصابة أو زيادة احتمالية الإصابة بحالة مرضية
- فقط في الحالات المؤكدة للإصابة أو زيادة احتمالية الإصابة بحالة مرضية ويمكن علاجها
- لا يجب الاتصال بي بأي حال من الأحوال

22. في حال أردت أن تسحب موافقتك على الإشتراك ببنك حيوي، إلى أي مما يلي برأيك يجب أن يؤدي انسحاب الموافقة؟ ((خيار واحد فقط *

*Mark only one oval.*

- التخلص من العينة فقط
- حذف البيانات فقط
- حذف البيانات والتخلص من العينة
- حذف هوية صاحب العينة والبيانات كلياً مع احتمالية استخدام العينة والبيانات في بحث جديد

شكراً على مشاركتك معنا. بإمكانك التعليق على الدراسة أدناه.

23.

This content is neither created nor endorsed by Google.

Google Forms
